# An Account of Two Cases of Fever, Induced by the Bite of a Venomous Insect, and Cured by Copious Depletion

**Published:** 1808-03

**Authors:** Samuel K. Jennings

**Affiliations:** Virginia


### *An Account of two Cases of Fever, induced by the Bite of a venomous Insect, and cured by copious Depletion.* By Dr. Samuel K. Jennings, of Virginia.

### Case I.

Mrs. Owen was bitten by an insect supposed to be a spider. About sunset she felt a peculiar sensation in the skin over the right hypochondrium. On examination, a small wound was discovered which had a double puncture, surrounded by a white elevated circle of about one inch in diameter. In a few minutes she perceived a pain commencing in the wound; thence quickly passing into her loins, and, in a very short time extending itself universally over the whole system. She complained of the pain being in her head, loins, hips, shoulders, and then again in her head, with irregular alternation, and all in the space of half a minute. It seemed impossible for her to rest a moment in any attitude. In a word, I could conceive of nothing short of the rack which could exceed her distress. Various nostrums were tried in vain. About midnight she was bled fourteen ounces. This evacuation afforded a momentary relief. But in a short time the violent lent symptoms returned, and continued without mitigation till ten o'clock the next day, at which time I arrived. Her pulse was as if natural. But the violence of the pain was such, that I suspected a degree of depression, and proceeded to take 14 oz. of blood. Her pulse seemed to yield a little, and although I had adopted that excellent opinion of Dr. Rush, "that fever is an unit," yet the novelty of the case led me to act rather feebly.

Supposing, however, that the continuance of the pain, might in part be owing to the retention of some improper matter in the intestines, I gave her 60 grs. of jalap, intending a speedy evacuation. After waiting two hours for the operation, I despaired of the effect, and ascribed the failure to the presence of that kind of torpor of the intestines, which attends highly inflammatory and malignant fever. Supported in this opinion by the idea of "the unity of disease," at twelve o'clock I drew 12 oz. of blood. Thinking that a dryness of the intestines might, in part, have prevented the operation of the jalap, about one o'clock I gave her a double portion of an infusion of senna, made considerably dilute. Waited till four o'clock for the operation; but in vain. Being now still more suspicious and even confident of a depressed state of the blood-vessels, I took eight ounces of blood. In the space of half an hour, the arterial action increased to such a pitch, as to equal any I ever saw of recent pleurisy. Immediately I proceeded with confidence and drew 16 oz. more of blood. The happiest effects were the consequence. The pains nearly subsided, and the pulse became soft, free, and very nearly natural. At six o'clock I gave her eight grains of calomel. She dropped into a disturbed kind of sleep, and rested indifferently till eleven o'clock; at which time the calomel excited nausea, terminating in a catharsis, and operated as usual. After the operation, the patient rested pleasantly till morning, and awoke quite well, except only that there was a moderate degree of debility. Nothing further was done, and in a few days she recovered her ordinary health.

In this case 64 oz. of blood were taken from the patient in the course of 20 hours. And prior to these evacuations, cathartic medicines, given in double and even in quadruple portions, would produce no effect. It is remarkable, that the calomel last given, operated no sooner, nor in a more copious manner, than a similar dose in ordinary cases would have done, without the addition of jalap, or infusion of senna.

CASE II.

### Case II.

Mrs. Brown was bitten by an insect, on the top of the foot. At the first it gave no pain; but, in about 15 minutes a peculiar painful sensation ascended up the affected limb, and spread itself over the whole system. The symptoms so nearly resembled the case of Mrs. Owen, that I have no doubt the same species of spider had produced them. The bite was first perceived in the evening. Her pain through the night was excruciating; insomuch, that she described it, as by far exceeding the most violent pains of parturition. All the remedies of the neighbourhood were tried in vain. The next morning I was sent for, but was absent. By the advice of one of my pupils, however, the messenger returned, and had her bled about 14 oz. at mid-day. As in case I. the blood-letting afforded relief for a short time. But the violent symptoms soon returned, and continued till about sunset, when I arrived to her relief. Finding her pulse very tense and active, I took from her 14 oz. of blood. Her pulse became softer, and her pains abated. In the course of half an hour, however, the action of the artery again increased. Emboldened by the success which had attended a similar practice in the former case, I proceeded without hesitation to repeat the blood-letting and took about 12 oz. The effects were as at the first, and the relief afforded was a little more complete, and of longer duration. But in less than one hour her pulse became more tense than at the first, and the pains were considerably increased. The blood-letting of course was again repeated, to the amount of 10 oz. The same good effects again followed the evacuation, and were again, still more permanent. In the course of an hour, the arterial action and pains were again becoming violent. Believing the stimulus of the venom to be more than sufficient to counteract the debility, which must certainly have followed such an amazing loss of blood in any ordinary case, and being still confident of success, I ventured to take 14 oz. more of blood. The effects were now very considerable. She complained of faintness, on being raised up in her bed; still, however, she was not without some degree of pain. She was then directed to make a free use of an infusion of senna, to which was added, a portion of tartarized antimony, until it should produce a considerable evacuation. As in case I. when the catharsis was complete, her disease disappeared, and nothing farther was done. In this case 64 oz. of blood were taken within within the space of nine hours, and 50 oz. between the hours of six and nine o'clock in the morning.

Do not these two cases strongly argue, that "fever is an unit," by whatever cause it may be induced?

Is it not highly probable, that a similar practice would relieve the painful fever, consequent on the bite of a snake?

I think it would be proper, in treating the snake bite, not only to introduce general, but also topical blood-letting. It may be observed, that the venom which was introduced into the system by the insects, in the cases of Mrs. Owen and Mrs. Brown, excited little, if any pain or swelling in the part first affected. It would seem that the virus is not perceived until it has spread its baneful powers over the whole system. But it is well known, that the venom of the rattlesnake gives immediate alarm to the surrounding vessels, and there is instantaneous resistance made to its progress, as is evident from the speedy pain and circumscribed swelling, which take place. It would therefore be proper to extend the general blood-letting according to general appearances, and by scarifications and cupping, to circumvent the topical affection. As the swelling and pain progress, the topical blood-letting should be proportionally extended.

That the two cases here described were the effects of the spider's bite, I have no doubt. Several cases have occurred within the bounds of my acquaintance, in which, the little venomous agent was seen to inflict the wound, or perceived immediately on having done the execution. In all these cases the effects were so entirely similar, that by every principle of analogy, it must be admitted, that all were produced by similar causes.

Do not the symptoms as described in case I. considerably resemble many cases of the yellow malignant fever which appeared in Philadelphia, in 1793?

P. S. The case of Mrs. Owen occurred in the autumn of 1805; that of Mrs. Brown about the 1st. of October, 1806.

